# Transcriptomic characteristics and impaired immune function of patients who retest positive for SARS-CoV-2 RNA

**DOI:** 10.1093/jmcb/mjab067

**Published:** 2021-10-23

**Authors:** Dongyao Wang, Dong Wang, Min Huang, Xiaohu Zheng, Yiqing Shen, Binqing Fu, Hong Zhao, Xianxiang Chen, Peng Peng, Qi Zhu, Yonggang Zhou, Jinghe Zhang, Zhigang Tian, Wuxiang Guan, Guiqiang Wang, Haiming Wei

**Affiliations:** 1 Division of Life Sciences and Medicine, Department of Hematology, The First Affiliated Hospital of University of Science and Technology of China, University of Science and Technology of China, Hefei 230001, China; 2 Institute of Immunology and the CAS Key Laboratory of Innate Immunity and Chronic Disease, School of Basic Medicine and Medical Center, University of Science and Technology of China, Hefei 230001, China; 3 Hefei National Laboratory for Physical Sciences at Microscale, University of Science and Technology of China, Hefei 230001, China; 4 Department of Laboratory Medicine, Tongji Hospital, Tongji Medical College, HuaZhong University of Science and Technology, Wuhan 430030, China; 5 Department of Infectious Diseases, Peking University First Hospital, Beijing 100034, China; 6 Department of Tuberculosis, Wuhan Pulmonary Hospital, Wuhan 430030, China; 7 Center for Emerging Infectious Diseases, Wuhan Institute of Virology, Chinese Academy of Sciences, Wuhan 430071, China; 8 Peking University International Hospital, Beijing 100034, China

**Keywords:** COVID-19, CD8^+^, T cells, NK cells, RTP patients, immune function

## Abstract

Coronavirus disease 2019 (COVID-19), caused by severe acute respiratory syndrome coronavirus 2 (SARS-CoV-2) infection, has become a global public health crisis. Some patients who have recovered from COVID-19 subsequently test positive again for SARS-CoV-2 RNA after discharge from hospital. How such retest-positive (RTP) patients become infected again is not known. In this study, 30 RTP patients, 20 convalescent patients, and 20 healthy controls were enrolled for the analysis of immunological characteristics of their peripheral blood mononuclear cells. We found that absolute numbers of CD4^+^ T cells, CD8^+^ T cells, and natural killer cells were not substantially decreased in RTP patients, but the expression of activation markers on these cells was significantly reduced. The percentage of granzyme B-producing T cells was also lower in RTP patients than in convalescent patients. Through transcriptome sequencing, we demonstrated that high expression of inhibitor of differentiation 1 (*ID1*) and low expression of interferon-induced transmembrane protein 10 (*IFITM10*) were associated with insufficient activation of immune cells and the occurrence of RTP. These findings provide insight into the impaired immune function associated with COVID-19 and the pathogenesis of RTP, which may contribute to a better understanding of the mechanisms underlying RTP.

## Introduction

Coronavirus disease 2019 (COVID-19) is caused by severe acute respiratory syndrome coronavirus 2 (SARS-CoV-2) infection. Globally, COVID-19 has spread to >200 countries, areas, or territories, and >180 million COVID-19 diagnoses worldwide have been reported to the World Health Organization (Wen et al., 2020; Yang et al., 2020; Calisher et al., 2021; Dai and Gao, 2021). Most hospitalized COVID-19 patients recover, but some retest positive (RTP) for SARS-CoV-2 again after recovery. Several studies have retrospectively analyzed the clinical and immunological characteristics of these patients as well as patients with severe COVID-19 (Tang et al., 2020; Wang et al., 2020a; Zheng et al., 2020a; Ni et al., 2021; Rha et al., 2021). It has been reported that the time from the first SARS-CoV-2 RNA negative to the re-positive was 20.6 ± 17.4 days, and it would remained positive for longer than 1 month (An et al., 2020; Xing et al., 2020; Zhao et al., 2021). However, the cause of RTP is still not known, which hampers the development of effective strategies for the treatment and care of patients who exhibit RTP. Therefore, discovering what causes RTP is very important. 

During viral infections, the immune response of the host is considered to determine the clinical outcome (Yang et al., 2020). We previously demonstrated that an excessive immune response by pathogenic T cells and inflammatory monocytes may be associated with severe lung disease (Fu et al., 2020; Xu et al., 2020). Wang et al. (2020a) showed that patients with severe COVID-19 had much higher levels of tumor necrosis factor-alpha (TNF-α), interleukin-2 (IL-2), and IL-10 in plasma and significantly higher expression of the activation marker human leukocyte antigen-DR isotype on T cells than patients with mild COVID-19. Increased plasma levels of macrophage colony-stimulating factor, granulocyte colony-stimulating factor, and interferon (IFN)-induced transmembrane protein 10 (IFITM10) are reported closely associated with lung-injury Murray score in COVID-19 patients (Liu et al., 2020). The immune response in RTP patients is not completely understood, and few studies have compared key changes in immune-cell subsets and their status in RTP patients and convalescent patients. Such comparisons may yield important insights into the mechanisms of the occurrence of RTP (Lu et al., 2020).

Inhibitor of differentiation 1 (ID1) promotes the growth, angiogenesis, and metastasis of tumor cells (Papaspyridonos et al., 2015; Castañón et al., 2017; Jin et al., 2018). ID1 expression can be induced by transforming growth factor-beta (TGF-β). High ID1 expression is reported significantly correlated with reduced overall survival in lung cancer patients (Lasorella et al., 2014), but the function of ID1 in COVID-19 patients is not known. Type I interferon (IFN-I) is considered to be the first line of host defense against viral infections and is a crucial component of innate immune responses (Chang et al., 2017; Neil et al., 2019; Tan et al., 2019; Wang et al., 2020b, c). It induces expression of a series of IFN-stimulated genes (ISGs), which are the main effectors of IFN-mediated antiviral responses (Schoggins, 2019; Lei et al., 2020). Notably, however, ISG expression in the peripheral blood mononuclear cells (PBMCs) of RTP patients and convalescent patients is poorly understood. Moreover, whether ISG expression influences immune-cell function in patients with COVID-19 is not known.

In the current study, flow cytometry and RNA sequencing were used to comprehensively investigate the immune features of PBMCs in RTP patients and convalescent patients. T cells and natural killer (NK) cells were weakly activated in RTP patients compared with convalescent patients. In RTP patients, the expression of some immunosuppressive genes, such as *LAG3* and *ID1*, increased markedly, whereas the expression of several ISGs decreased markedly. The results of the study provide new insights into how RTP may occur.

## Results

### Immune characteristics of RTP patients

When readmitted to hospital, RTP patients did not exhibit any obvious clinical symptoms or infectivity. They did not exhibit fever. Of 30 RTP patients, 8 (26.7%) exhibited upper respiratory symptoms such as coughing, 3 (10.0%) exhibited chest tightness, and 4 (13.3%) exhibited fatigue (Supplementary Table S3). Next, the immune and serological characteristics of RTP patients were investigated. Fresh PBMCs were isolated from 30 RTP patients immediately after diagnosis, 20 convalescent patients, and 20 healthy controls. Laboratory tests were then conducted on serum to determine routine biochemistry, C-reactive protein (CRP), and lactate dehydrogenase. In addition, laboratory tests were also performed on whole blood to determine hematocrit and hemoglobin.

**Figure 1:**
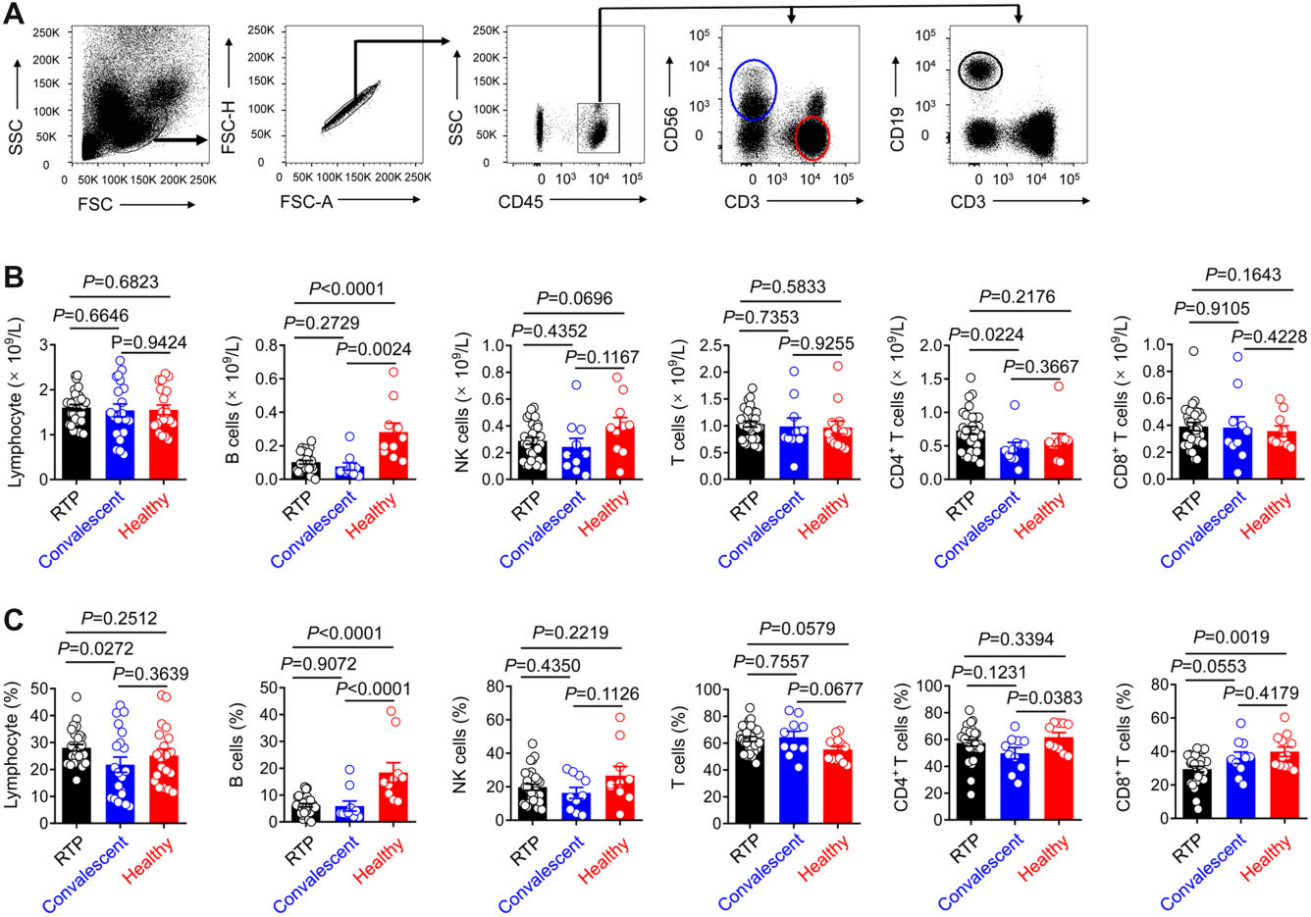
Immunological characteristics of RTP patients. (**A**) Gating strategy for CD3^+^ T cells, B cells, and NK cells from PBMCs. (**B** and **C**) The absolute numbers (**B**) and frequencies (**C**) of lymphocytes, B cells, NK cells, T cells, CD4^+^ T cells, and CD8^+^ T cells as determined by flow cytometry in RTP patients (*n *=* *30), convalescent patients (*n *=* *20), and healthy controls (*n *=* *20). Each dot represents one sample. Data were analyzed by two-way ANOVA. Data are presented as mean ± SD.

It was reported that a decrease in the number of T cells—particularly CD4^+^ T cells—is a hallmark of COVID-19 (Wiersinga et al., 2020; Zhu et al., 2020). In the present study, there were no significant differences in the numbers of NK cells, T cells, CD4^+^ T cells, or CD8^+^ T cells among the three groups, but there was a significant reduction in the number of B cells in RTP patients compared to healthy controls (Figure 1). There were no significant differences in the numbers of monocytes or neutrophils among RTP patients, convalescent patients, and healthy controls (Supplementary Figure S1A and B). CRP levels were higher in RTP patients than in convalescent patients and healthy controls. Levels of hemoglobin and hematocrit in RTP patients were much higher than those in convalescent patients. There were no significant differences in lactate dehydrogenase levels among the three groups. Collectively, these results indicated that although RTP patients retested positive for SARS-CoV-2 RNA, the number of immune cells in their peripheral blood remained stable.

### T cells are weakly activated in RTP patients

**Figure 2:**
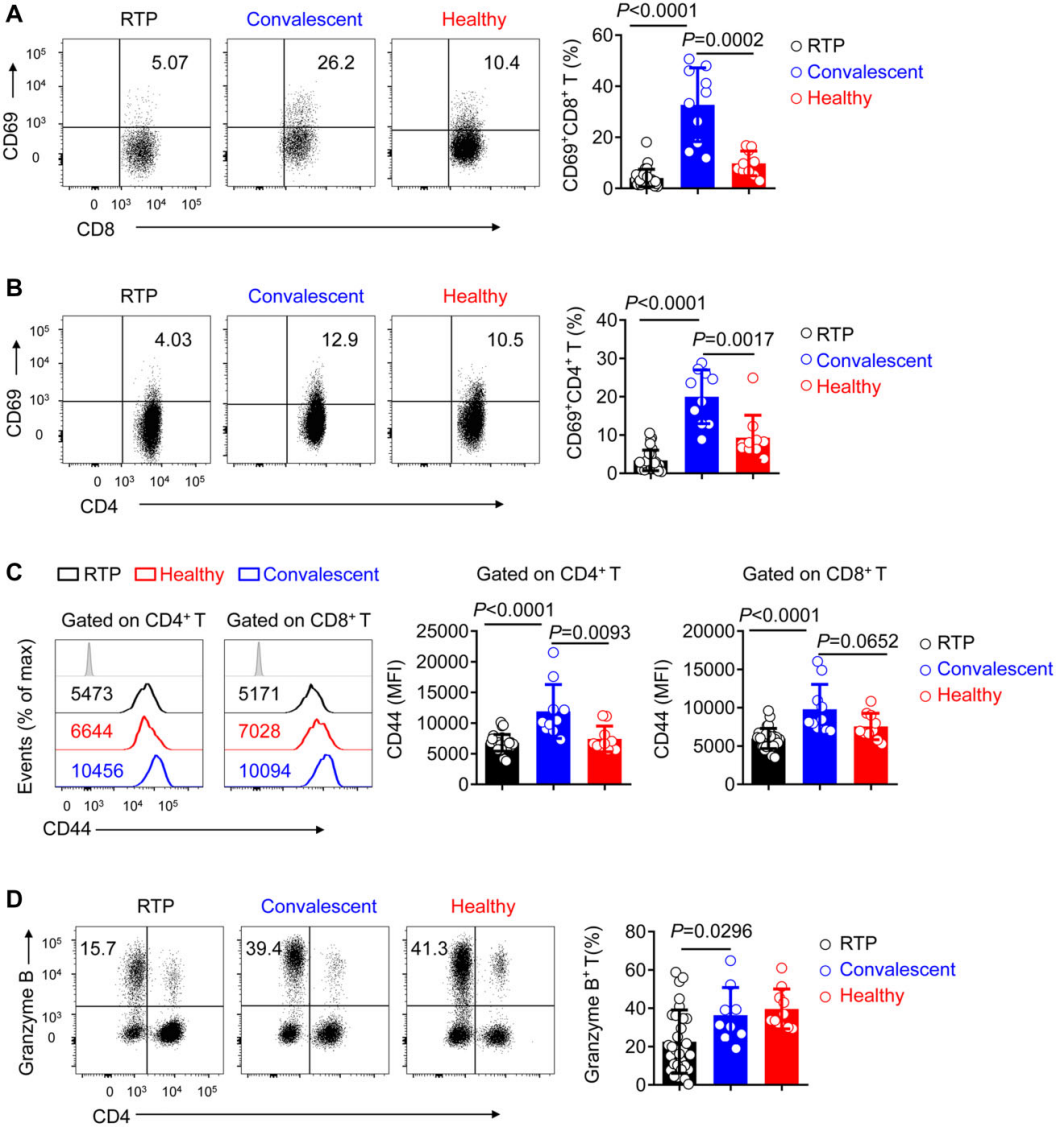
T cells maintain low activation in RTP patients. (**A** and **B**) Representative density plots and percentage statistics calculated for CD69 expression in gated CD45^+^CD3^+^CD8^+^ T cells (**A**) and CD45^+^CD3^+^CD4^+^ T cells (**B**) isolated from the blood of RTP patients, convalescent patients, and healthy controls. (**C**) Flow cytometry of CD44 expression in gated CD45^+^CD3^+^CD4^+^ T cells and CD45^+^CD3^+^CD8^+^ T cells isolated from the peripheral blood of RTP patients, convalescent patients, and healthy controls. (**D**) Representative density plots and percentage statistics calculated for granzyme-B expression in gated CD45^+^CD3^+^ T cells isolated from the peripheral blood of RTP patients, convalescent patients, and healthy controls. *n *=* *30 for RTP patients, *n *=* *10 (randomly selected from 20 samples) for convalescent patients, and *n *=* *10 (randomly selected from 20 samples) for healthy controls. Data were analyzed by two-way ANOVA. Data are presented as mean ± SD.

The activation and effector functions of T cells and NK cells are crucial for viral clearance (Sivori et al., 2019; Demaria et al., 2020; Sung et al., 2021). To further investigate why RTP patients tested positive again, we attempted to determine whether they exhibited increases in the expression of immunosuppressive molecules. In CD4^+^ T cells and CD8^+^ T cells, there were no significant differences in the expression of PD-1 or TIM-3 among RTP patients, convalescent patients, and healthy controls (Supplementary Figure S2A and B). TIM-3 expression on NK cells was not upregulated in RTP patients (Supplementary Figure S2C). Because T-cell activation was limited, expression levels of CD69 and CD44 were measured. CD69 expression and the mean fluorescence intensity (MFI) of CD44 in CD4^+^ T cells and CD8^+^ T cells were significantly lower in RTP patients than in convalescent patients (Figure 2A–C). T cells from RTP patients exhibited reduced granzyme B expression, suggesting impaired function (Figure 2D). Collectively, these results indicated that although there was no marked change in the number of T cells, weakly activated T cells in RTP patients may contribute to the obstruction of SARS-CoV-2 clearance.

### Insufficient activation of NK cells in RTP patients

**Figure 3:**
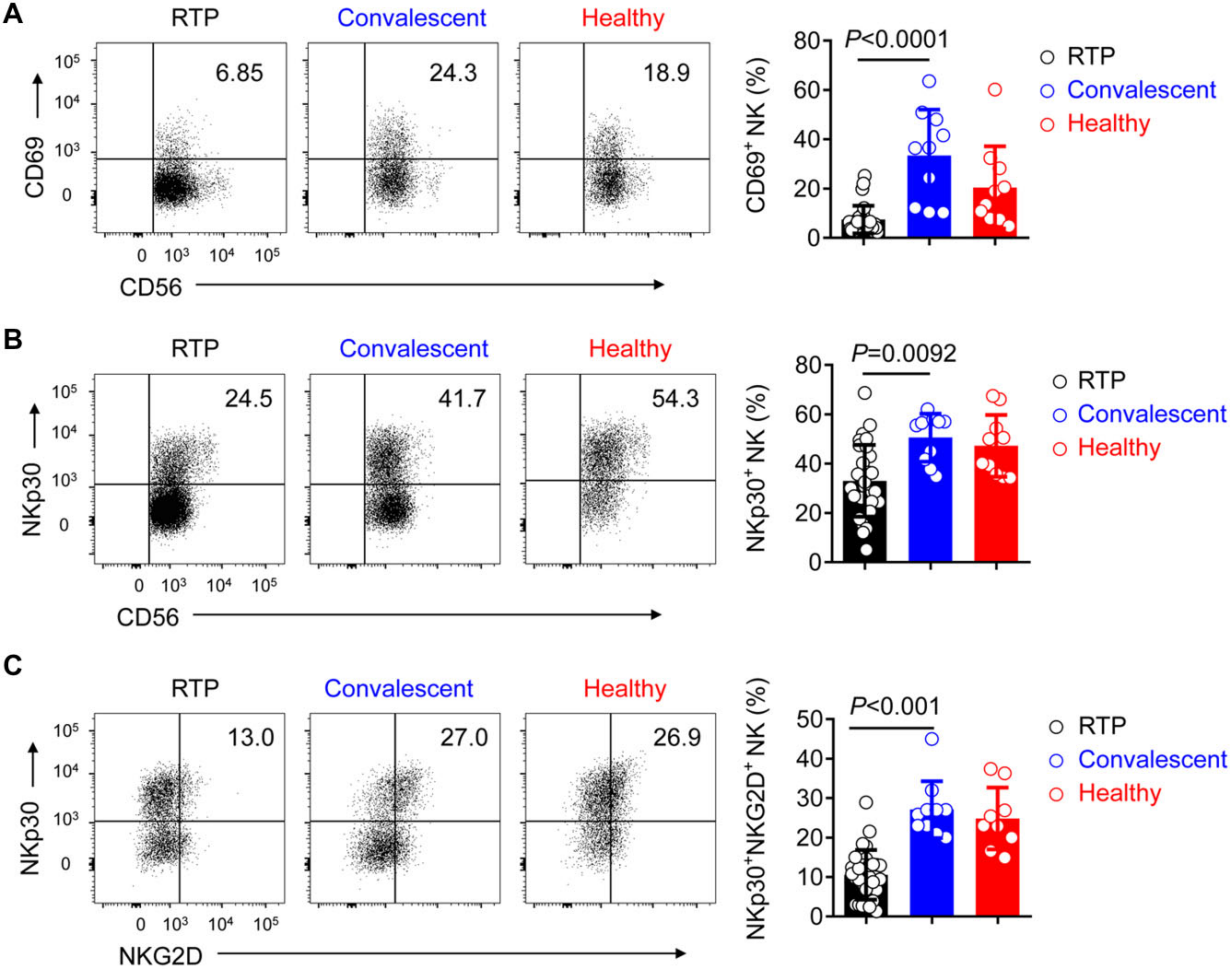
NK cells maintain low activation in RTP patients. (**A** and **B**) Representative density plots and percentage statistics calculated for the expression of CD69 (**A**) and NKp30 (**B**) in gated CD45^+^CD3^−^CD56^+^ NK cells isolated from the peripheral blood in RTP patients, convalescent patients, and healthy controls. (**C**) Representative density plots and percentage statistics calculated for the co-expression of NKp30 and NKG2D in gated CD45^+^CD3^−^CD56^+^ NK cells isolated from the peripheral blood of RTP patients, convalescent patients, and healthy controls. *n *=* *30 for RTP patients, *n *=* *10 (randomly selected from 20 samples) for convalescent patients, and *n *=* *10 (randomly selected from 20 samples) for healthy controls. Data were analyzed by two-way ANOVA. Data are presented as mean ± SD.

Cells of the innate immune system such as NK cells play an important part in defense against viral infection. SARS-CoV-2 infection reportedly impedes NK-cell function (Maucourant et al., 2020; van Eeden et al., 2020; Zheng et al., 2020b), and thus in the current study, the activation of NK cells was investigated in RTP patients. Consistent with the results of T cell analysis, NK cells exhibited significantly reduced expression of CD69 and NKp30, which is reportedly associated with NK-cell cytotoxicity (Shen et al., 2016), in RTP patients but not in convalescent patients. The percentage of NKp30^+^NKG2D^+^ NK cells was markedly reduced in RTP patients (Figure 3A–C). These findings suggest that, in addition to T cells, NK cells were also not fully activated, and this may be associated with the redetection of SARS-CoV-2 RNA.

### Increased expression of ID1 in RTP patients

**Figure 4:**
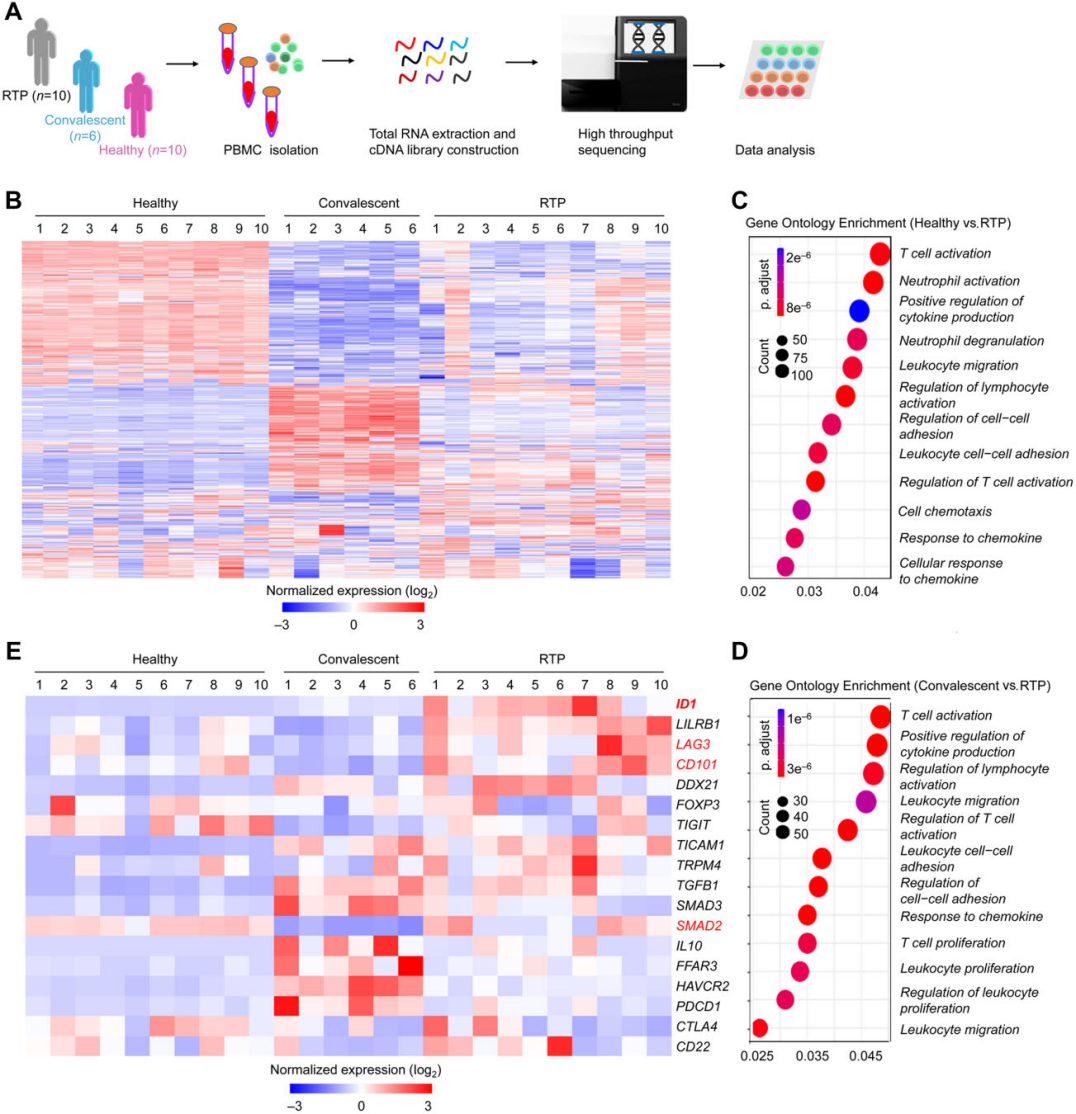
Leukocytes express negative immunomodulatory molecules and *ID1* at high levels in RTP patients. (**A**) Scheme showing the protocol for isolation of PBMCs from healthy controls (*n *=* *10), convalescent patients (*n *=* *6), and RTP patients (*n *=* *10) with COVID-19. Human samples from each group were randomly selected. RNA sequencing was done using Seq-Well. (**B**) The top 500 genes with differential expression (>2-fold) in RTP patients compared with that in convalescent patients and healthy controls were selected for heatmap analyses. Each column depicts one sample. (**C**) Enrichment analyses of DEGs were done (using the GO database) to evaluate enriched biological processes between RTP patients and healthy controls. Enrichment of regulation of lymphocyte activation-related biological processes was found. (**D**) Enrichment analyses (using the GO database) of the negative regulation of immune-system processes between RTP patients and convalescent patients. Enrichment of regulation of lymphocyte activation-related biological processes was found. (**E**) Heatmap showing the normalized expression of negative immunomodulatory genes in RTP patients compared with that in convalescent patients and healthy controls.

Insufficient activation of T cells and NK cells was associated with redetection of SARS-CoV-2 RNA. Bulk RNA sequencing was used to compare the transcriptomes of PBMCs from RTP patients (*n *=* *10), convalescent patients (*n *=* *6), and healthy controls (*n *=* *10) and assess relative gene expression (Figure 4A). The transcriptional profiles of PBMCs from RTP patients differed from those of healthy controls and convalescent patients. A total of 2105 genes exhibited upregulated expression and 1988 genes exhibited downregulated expression in RTP patients compared with convalescent patients. A total of 2858 genes exhibited upregulated expression and 1460 genes exhibited downregulated expression in RTP patients compared with healthy controls. In convalescent patients, 4077 genes exhibited upregulated expression and 3063 genes exhibited downregulated expression compared with healthy controls (Figure 4B;  Supplementary Figure S3A). Enrichment analyses using the Gene Ontology (GO) database revealed altered immunological processes, including regulation of lymphocyte activation and cytokine production (Figure 4C and D). By comparing RTP patients vs. healthy controls, RTP patients vs. convalescent patients, and convalescent patients vs. healthy controls, we found that negative regulators of immune system signaling and lymphocyte activation signaling were enriched in PBMCs from RTP patients (Supplementary Figure S3B–E).

Next, data from the current study were compared with the results of other studies involving patients with active SARS-CoV-2 infection. Firstly, percentages of different PBMC subpopulations in RTP patients (reported in this study) were compared with those in moderate COVID-19 patients and severe COVID-19 patients (reported by Zhou et al., 2021). Results showed that, in RTP patients, the percentage of B cells was significantly lower and the percentages of NK cells and T cells were significantly higher (Supplementary Figure S4A). Secondly, RNA sequencing data were compared with those from Yao et al. (2021). We found that the transcriptional profiles of PBMCs from RTP patients were different from those of patients with moderate or severe COVID-19. We demonstrated the downregulation of *IFITM10*, *CH25H*, *NCR3*, and *KLRK1* and the upregulation of *ID1* in the PBMCs from RTP patients compared with that from patients with moderate or severe COVID-19 (Supplementary Figure S4B and C). Relative MFIs (normalized to the mean MFI in healthy controls) of CD69 and CD44 on CD4^+^ T cells, CD8^+^ T cells, and NK cells were compared with those from Zhou et al. (2021). The relative MFIs of CD69 and CD44 in CD4^+^ T cells, CD8^+^ T cells, and NK cells were significantly lower in RTP patients than in patients with moderate COVID-19 and severe COVID-19 (Supplementary Figure S4D).

**Figure 5:**
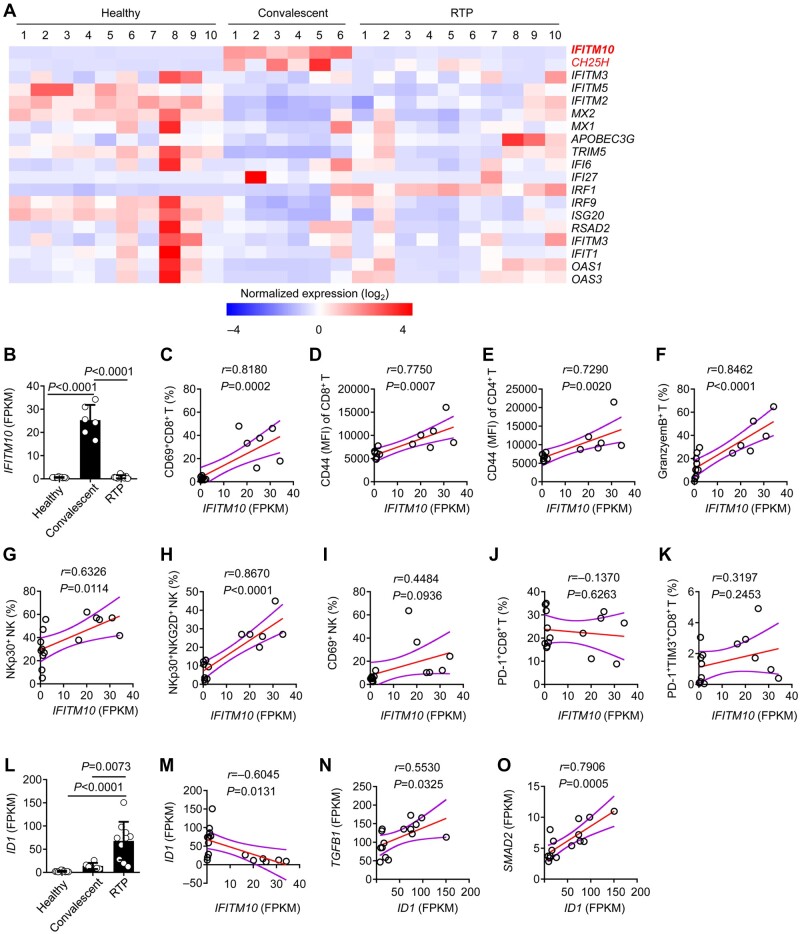
Leukocytes express *IFITM10* at low levels in RTP patients. (**A**) Heatmap of IFN-stimulated genes with differential expression in RTP patients compared with that in convalescent patients and healthy controls. (**B**) Fragments per kilobase of exon model per million mapped fragments (FPKM) of *IFITM10* in RTP patients compared with that in convalescent patients and healthy controls. (**C**–**K**) Spearman’s rank correlation coefficient comparing FPKM of *IFITM10* and the proportion of the indicated molecules in RTP patients and convalescent patients. The Spearman correlation coefficient (*r*) and *P*-value are shown. (**L**) FPKM of *ID1* in RTP patients compared with that in convalescent patients and healthy controls. (**M**) FPKM of *IFITM10* shows a negative correlation with FPKM of *ID1.* (**N**) FPKM of *IFITM10* shows a positive correlation with FPKM of *TGFB1.* (**O**) FPKM of *IFITM10* shows a positive correlation with FPKM of *SMAD2.* The Spearman correlation coefficient (*r*) and *P*-value are shown.

By performing differentially expressed gene (DEG) analysis, we found that many immunosuppressive and exhaustion-related genes such as *LAG3*, *CD101*, *SMAD2*, and *ID1*, which was one type of the mediators of tumor progression (Perk et al., 2006), were highly expressed in PBMCs from RTP patients (Figures 4E and 5L). Collectively, the above results revealed differences in transcriptional profiles in PBMCs from RTP patients, convalescent patients, and healthy controls. Specifically, PBMCs from RTP patients mainly showed insufficient activation of immune cells.

### Expression of ID1 was negatively correlated with IFITM10 expression

IFN-I is a vital component of innate immune responses. It is considered the first line of host defense against viral infections. SARS-CoV-2 is sensitive to IFN-β treatment (Lei et al., 2020; Mantlo et al., 2020). Once secreted, IFN-I-induced expression of ISGs is vital for the control of infectivity. RNA sequencing data showed that, compared to convalescent patients, there was a substantially attenuated expression level of *CH25H* and *IFITM10* in RTP patients. To be specific, in RTP patients, *CH25H* expression level was only 20% of that in convalescent patients, and *IFITM10* expression level was only 3% of that in convalescent patients (Figure 5A and B). However, expression levels of some other ISGs such as *MX1*, *IRF9*, and *IFITM3* were not significantly lower in RTP patients than in convalescent patients.

IFITM proteins were reported to inhibit early steps in the lifecycles of various viruses (Diamond and Farzan, 2013; Bailey et al., 2014; Yánez et al., 2020). Therefore, we investigated whether low *IFITM10* expression was correlated with the observed impaired antiviral effects exerted by T cells and NK cells. *IFITM10* expression level was positively correlated with the expression level of CD69, CD44, NKp30, and granzyme B (Figure 5C–K). The expression level of *ID1* was negatively correlated with the expression level of *IFITM10* but positively correlated with that of *TGFB1* and *SMAD2*, which plays central roles in immune suppression and regulates the effector functions of many immune-cell types (Figure 5L–O).

Huang et al. (2020) reported that patients with COVID-19 had increased serum levels of proinflammatory cytokines such as IL-2, IL-6, IL-7, IL-10, TNF-α, monocyte chemoattractant protein 1, granulocyte colony-stimulating factor, IFN-γ-inducible protein 10, and macrophage inflammatory protein 1. This was observed particularly in patients admitted to intensive care and indicated that a ‘cytokine storm’ had occurred (Fu et al., 2020; Huang et al., 2020). In the present study, some inflammation-related genes such as *IL-1B*, *IL-4*, and *TNF* exhibited high expression in PBMCs from RTP patients even though severe pneumonia was not observed clinically (Supplementary Figure S5). Collectively, these findings suggest that attenuated expression of ISGs in RTP patients may be related to the observed inadequate antiviral activity, which further increases the likelihood of redetection of SARS-CoV-2 RNA.

## Discussion

RTP patients have been identified in several studies, but the mechanisms underlying RTP occurrence are poorly understood. We suggest that insufficient immune system antiviral function may contribute to RTP. In the current study, in most RTP patients, neither T cells nor NK cells exhibited strong activation. Immunosuppressive genes and exhaustion-related genes such as *LAG3*, *CD101*, and *ID1* exhibited high expression in these patients. *IFITM10* expression was positively correlated with expression of markers of T-cell and NK-cell activation in patients. Collectively, these findings make three important contributions to elucidating the mechanisms underlying the occurrence of RTP.

First, routine laboratory tests were conducted to elucidate the immunological and serological characteristics of RTP patients. The number of immune cells in the peripheral blood of RTP patients remained stable compared with that in convalescent patients. T cells and NK cells play important roles in the host defense against viral infections (Shen et al., 2016; Kumar et al., 2018; Kurachi, 2019). Zhou et al. (2021) reported that in patients receiving intensive care the numbers of T cells, CD4^+^ T cells, and CD8^+^ T cells decreased dramatically. However, severe pneumonia was not observed in RTP patients, and the numbers of T cells, NK cells, monocytes, and neutrophils were not significantly reduced in the current study. Therefore, we surmised that other factors may mediate the occurrence of RTP.

Second, we provided evidence that insufficient activation of T cells and NK cells may play a part of role in the occurrence of RTP. Considerable research on the strong antiviral effects of human T cells and NK cells has been reported, and activation of these cells is a prerequisite for viral clearance (O'Brien and Finlay, 2019; Hao et al., 2021). Several studies have demonstrated that the T cells of COVID-19 patients progress from the prodromal stage to the symptomatic stage and exhibit dramatically increased expression of PD-1 and TIM-3 (Diao et al., 2020; Zheng et al., 2020a). Furthermore, excessive activation of T cells has been observed in patients with severe disease relative to patients with mild disease (Song et al., 2020; Wang et al., 2020a). In the present study, expression of PD-1 and TIM-3 did not differ significantly among RTP patients, convalescent patients, and healthy controls. Yao et al. (2021) demonstrated that COVID-19 patients with acute respiratory distress syndrome were in a state of ‘immune imbalance’ in which dysregulation of adaptive and innate immune responses may contribute to a more severe disease course. Notably, however, expression of several activation markers (i.e. CD69, CD44, NKp30, NKG2D, and granzyme B) was significantly reduced in RTP patients. These findings indicate that the insufficient activation of T cells and NK cells, not their exhaustion, is associated with the occurrence of RTP. There was also high expression of *IL-1B*, *IL-4*, and *TNF* in PBMCs from RTP patients in the current study. Wen et al. (2020) characterized transcriptional changes in PBMCs during the recovery stage of COVID-19 via single-cell RNA sequencing. They predicted that IL-1β may be one of the novel candidate target genes in an inflammatory storm. Du et al. (2021) reported that compared with children not suffering from pneumonia (manifested as asymptomatic and acute infection of the upper respiratory tract), children suffering from pneumonia exhibited higher serum levels of IL-4 and TNF-α. The present study indicated that there may be the potential for inflammation in RTP patients.

Third, we identified high *ID1* expression whereas low *IFITM10* expression in RTP patients. The reasons underlying the occurrence of RTP may be related to virological factors such as residual viral load, viral distribution, and intermittent viral release (Wölfel et al., 2020). ID1 is overexpressed in many tumor types and promotes tumor growth (Perk et al., 2006; Lasorella et al., 2014; Castañón et al., 2017). The loss of ID1 may lead to a decrease in TGF-β1 expression (Castañón et al., 2017). Therefore, *ID1* overexpression may be associated with suppressed antiviral immune function. ISGs exert multiple antiviral functions, many of which have not been fully described (Fan et al., 2020). Zhang et al. (2020) reported that most cell types in patients with COVID-19, including moderate, severe, and convalescent cases, exhibited a strong IFN-α response. IFITM-family proteins such as IFITM2/3 are crucial ISG products. IFITM proteins reportedly prevent infection during the early stages of lifecycles of several viruses. IFITM10 is the most highly conserved member of the IFITM family, but its function is unclear (Poddar et al., 2016). In the present study, the transcriptional profiles of PBMCs from RTP patients differed from those from convalescent patients and healthy controls. *ID1* exhibited significantly higher expression in RTP patients than in convalescent patients, whereas *IFITM10* exhibited dramatically lower expression. *IFITM10* expression was positively correlated with the expression of CD69, CD44, NKp30, and granzyme B but was negatively correlated with *ID1* expression. The expression of *ID1* was positively correlated with that of *TGFB1* and *SMAD2*. Collectively, these observations suggest a possible mechanism of the occurrence of RTP.

The present study had five main limitations. First, we did not obtain successive samples from RTP patients and convalescent patients. Second, we did not determine whether RTP patients had active infection. Third, while laboratory errors were carefully avoided, we could not exclude other reasons why patients who had recovered from COVID-19 again tested positive for SARS-CoV-2 RNA. Fourth, potential long-term residual viral loads in tissues (e.g. gut) may be associated with an increased risk of RTP (Wu et al., 2020), but we did not explore this factor in the current study. Fifth, owing to the limited amount of peripheral blood samples from RTP patients, only flow cytometry and bulk RNA sequencing were performed. We would have performed more experiments and provided more informative insight, but at present it is difficult to acquire peripheral blood samples from RTP patients. In addition, our laboratory does not meet Biosafety Level-3 Protection Standards. Therefore, further laboratory and epidemiological studies are required to determine the true causes of the occurrence of RTP. Collectively, the results of the present study reveal immune features of RTP patients and may advance understanding of the mechanisms involved in the occurrence of RTP in people who have recovered from COVID-19.

## Materials and methods

### Ethical approval of the study protocol

The study protocol was approved by the Ethics Committee of the First Affiliated Hospital of University of Science and Technology of China (2020-XG(H)-005; Hefei, Anhui Province) and Peking University First Hospital for Emerging Infectious Diseases (2020-Research-112; Beijing). Experiments were conducted in accordance with the ethical guidelines of the 1964 Declaration of Helsinki and its later amendments, the Principles of Good Clinical Practice, and guidelines set by the Chinese government.

### Study cohort

Samples of peripheral blood were collected from 30 RTP patients (16 males and 14 females; median age of 62 [42–78] years) and 20 convalescent patients (10 males and 10 females; median age of 66.7 [32–87] years) with COVID-19 at the Wuhan Pulmonary Hospital (Wuhan, Hubei Province) and E’zhou Central Hospital (E’zhou, Hubei Province), with the consent of all patients. According to the *Guidelines For The Diagnosis For Novel Coronavirus Pneumonia* published by the National Health Commission of the People’s Republic of China (An et al., 2020), all first-diagnosis cases of COVID-19 were confirmed on the basis of positive real-time reverse transcription–quantitative polymerase chain reaction (RT–qPCR) tests. The peripheral blood samples of 20 healthy controls (12 males and 8 females; median age of 67.1 [22–74] years) were collected from Tongji Hospital (Wuhan, Hubei Province) and the First Affiliated Hospital of University of Science and Technology of China (Hefei, Anhui Province).

### Clinical definitions

The criteria for hospital discharge of convalescent patients were (i) body temperature returned to normal for >3 days; (ii) initially diagnosed with COVID-19, but at least two consecutive SARS-CoV-2 RT–qPCR assays of sputum with a sampling interval of 24 h being negative (Cao et al., 2020; Li et al., 2020); (iii) respiratory symptoms improved noticeably; and (iv) significant absorption of pulmonary lesions upon pulmonary imaging. All discharged COVID-19 patients were isolated and continued to be observed for 14 days, with regular follow-up each week, and timely SARS-CoV-2 RNA testing was undertaken. Samples from convalescent patients were collected from the patients 2–4 weeks after they had been discharged from the hospital (Li et al., 2020), with negative RT-qPCR results for SARS-CoV-2 RNA (as in (ii) above) and no persisting COVID-19 symptoms.

‘RTP patients’ were defined as follows. (i) Discharged patients were initially diagnosed with COVID-19, in whom post-treatment SARS-CoV-2 RT–qPCR assays of respiratory specimens such as sputum or nasopharyngeal swabs had been negative for ≥2 consecutive tests (sampling time interval of at least 24 h). (ii) These patients continued to be isolated and closely observed for 14 days, then were followed up weekly, and subject to SARS-CoV-2 RNA detection timely. The discharged patients were subsequently followed up for at least additional 14 days after isolation (An et al., 2020). (iii) Then, ≥2 consecutive RT–qPCR assays of samples comprising nasopharyngeal swabs, throat swabs, or anal swabs with a sampling interval of 24 h turned positive at follow-up after discharge from hospital (Zhao et al., 2021), and the samples were immediately collected.

Neither RTP patients nor convalescent patients exhibited severe clinical symptoms. Detailed patient information is shown in Supplementary Table S1.

### Flow cytometry

PBMCs were isolated from peripheral blood by density gradient centrifugation using Ficoll-Paque media and stained with human monoclonal antibodies (as described in Supplementary Table S2). Before staining with antibodies, mouse serum was used to block the binding of non-specific Fc-receptors. For intracellular staining, fresh isolated leukocytes were stained for surface markers for 30 min at 4°C. Then, cells were fixed for 30 min at 4°C, permeabilized with a Foxp3/Transcription Factor Staining Buffer Set, and stained with intracellular antibodies for 1 h at 4°C. Data were collected using a flow cytometer (FCM LSR II; BD Biosciences) and analyzed with FlowJo (Tree Star).

### Transcriptome sequencing

Twenty-six PBMC samples from the three groups were subjected to transcriptome sequencing using the BGISEG platform at Beijing Genomics Institution (BGI). Briefly, 1 ml TRIzol^®^ Reagent (Invitrogen) was added to 2 × 10^6^ PBMCs from each sample and stored at −80°C. Total RNA was extracted and eluted in diethylpyrocarbonate-treated water. According to the standard BGI protocol, RNA libraries were prepared for transcriptome sequencing using the MGISEQ-2000 RS High Throughput (Rapid) Sequencing Reagent Kit. We used oligo(dT)-attached magnetic beads to purify messenger RNA (mRNA). Then, the purified mRNA was fragmented into small pieces with fragment buffer at the appropriate temperature. Next, first-strand complimentary DNA (cDNA) was generated using random hexamer-primed reverse transcription, followed by a second-strand cDNA synthesis. A-Tailing Mix and RNA Index Adapters were added by incubating to end repair. The cDNA fragments obtained from the previous step were amplified by PCR, and the products were purified by AMPure XP Beads and then dissolved in EB solution. We validated the product on a bioanalyzer (2100; Agilent Technologies) for quality control. The double-stranded PCR products from the previous step were heated, denatured, and circularized by the splint oligo sequence to obtain the final library. The single-strand circlular DNA was formatted as the final library. The latter was amplified with phi29 to make a ‘DNA nanoball’ (DNB), which had >300 copies of one molecule. Then, DNBs were loaded into a patterned nanoarray and 100-base pair-end reads were generated on the BGISEG platform. The sequencing data were filtered with SOAPnuke v1.5.2 by removing the reads: (i) containing a sequencing adapter; (ii) whose low-quality base (base quality ≤5) ratio was >20%; and (iii) whose unknown base (‘N’ base) ratio was >5%. Afterwards, clean reads were obtained and stored in FASTQ format. Then, clean reads were mapped to the reference genome using HISAT2 v2.0.4. Analyses of differential expression were done using DESeq2 v1.4.5 with Q ≤ 0.05.

‘DEGs’ were defined as those with a fold change ≥2.0 in expression between two groups and signal values higher than background signals. An adjusted *P*-value was used to determine DEGs. To obtain insights in the change of phenotype, enrichment analyses of DEGs were done using the GO (www.geneontology.org/) and Kyoto Encyclopedia of Genes and Genomes (KEGG; www.kegg.jp/) databases. The significance levels of terms and pathways were corrected with a rigorous threshold of Q (≤0.05) by Bonferroni correction. The *P*-value for each gene and gene set shown was significant. Raw RNA sequencing data have been deposited in the Gene Expression Omnibus Repository of the National Center for Biotechnology Information (accession number: GSE166253).

### Statistical analyses

Data are presented as mean ± standard deviation (SD). We undertook two-way analysis of variance (ANOVA) across multiple groups. Prism 6 (GraphPad) was used to determine significance. Statistical parameters are indicated in the figure legends. *P *<* *0.05 was considered significant.

## Supplementary material

Supplementary material is available at *Journal of Molecular Cell Biology* online.

## Supplementary Material

mjab067_Supplementary_Data
