# Supplementary material for: Transcriptomic characteristics and impaired immune function of patients who retest positive for SARS-CoV-2 RNA
Source: J Mol Cell Biol. 2021 Oct 23;13(10):748–59. doi: 10.1093/jmcb/mjab067 (PMC8574305; doi:10.1093/jmcb/mjab067)
Supplement: mjab067_Supplementary_Data [file mjab067_supplementary_data.zip › Supplementary material.pdf]

Supplementary Figures

Sfig.1

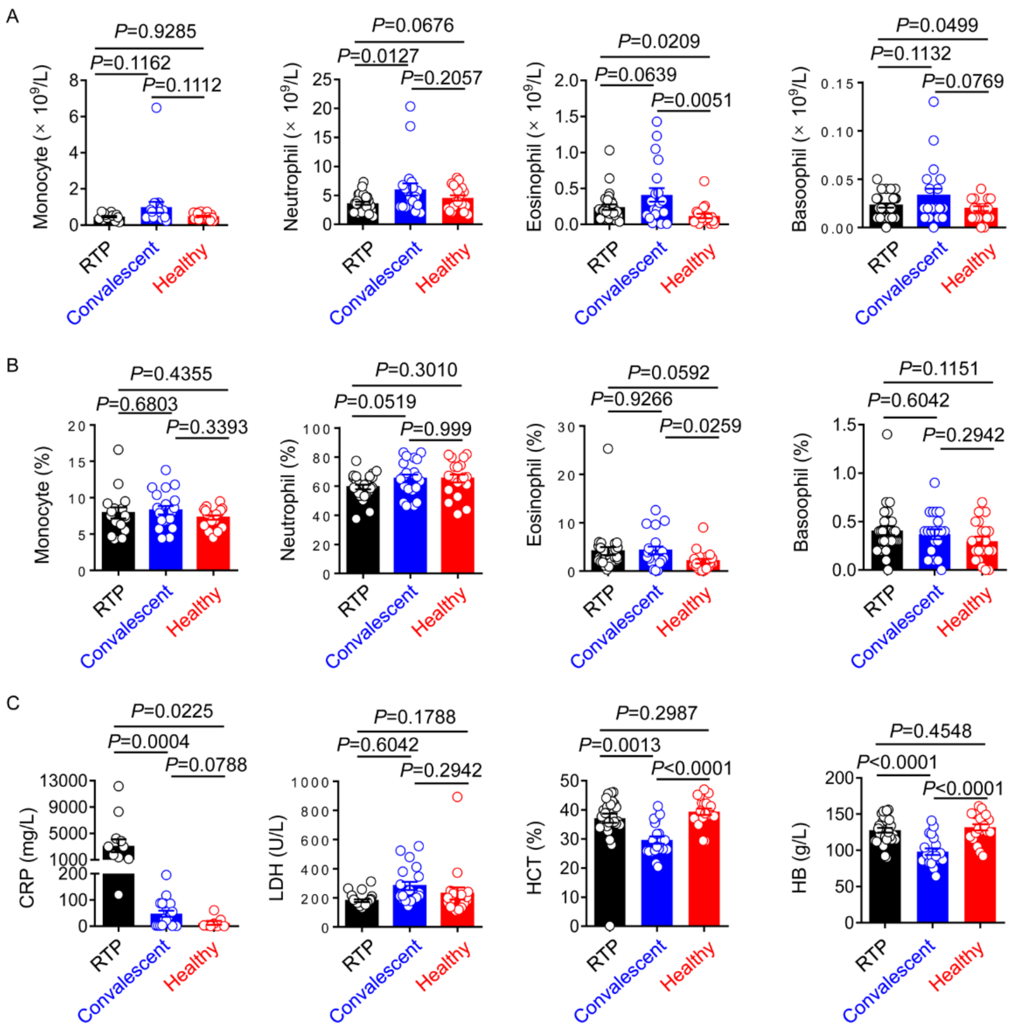

Supplementary Figure 1. Immunological and serological characteristics of RTP patients.

(**A**) The absolute number and (**B**) frequencies of monocytes, neutrophils, eosinophils, and basophils as determined by flow cytometry in healthy controls, convalescent patients, and RTP patients with COVID-19; n = 20–30.

(**C**) Quantification of CRP, LDH, percentage of HCT, and Hb in healthy controls, convalescent patients, and RTP patients with COVID-19; n = 20–30.

Data were analyzed by two-way ANOVA. $*P < 0.05$; $**P < 0.01$; $***P < 0.001$; $****P < 0.0001$. Data are presented as mean ± SD.

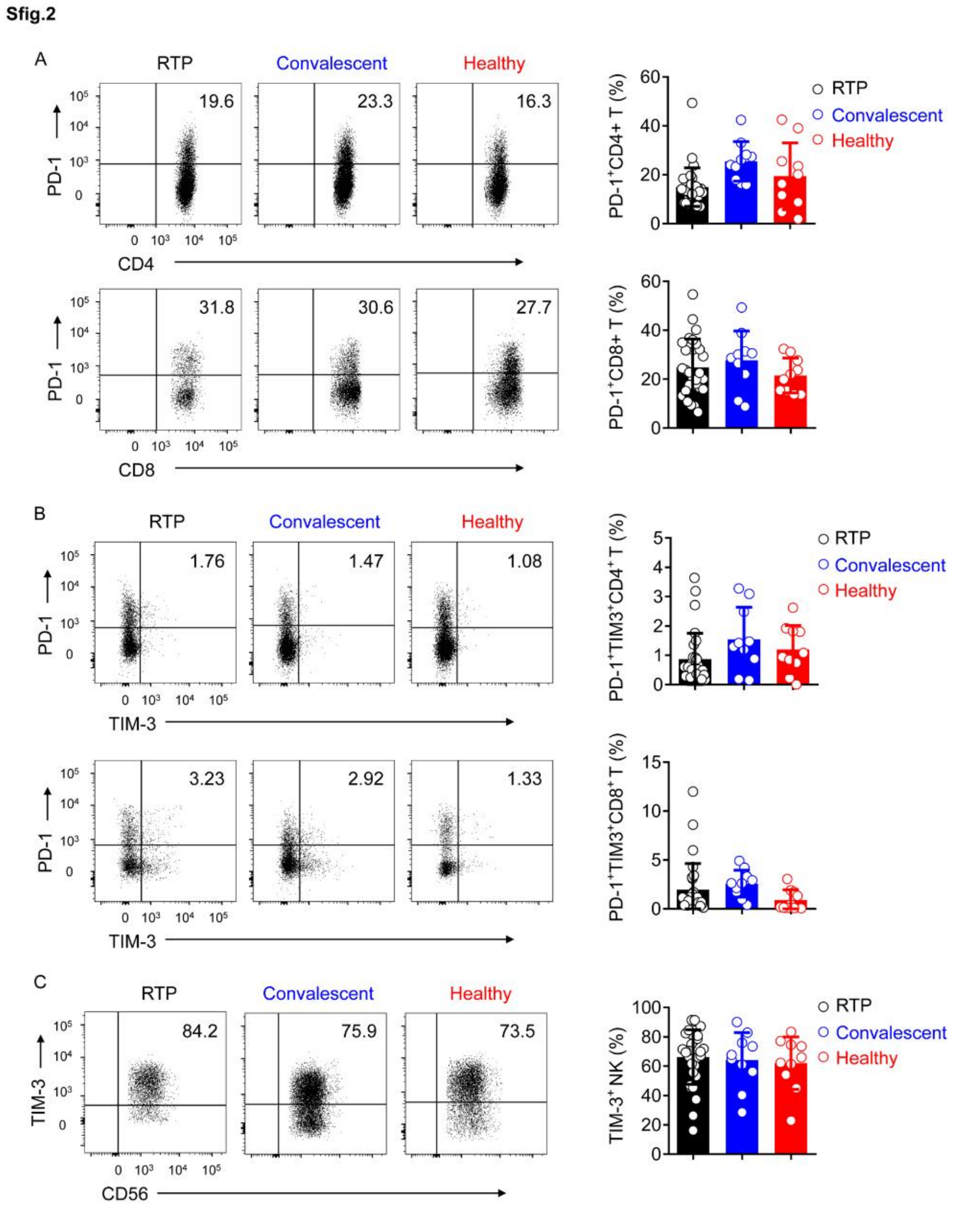

Supplementary Figure 2. The expression of PD-1 and TIM-3 did not increase in RTP patients.

(**A**) Representative density plots and percentage statistics calculated for PD-1 expression in gated $CD4^{+}$T cells and $CD8^{+}$T cells isolated from the peripheral blood of RTP patients, convalescent patients, and healthy controls.

(**B**) Representative density plots and percentage statistics calculated for the co-expression of PD-1 and TIM-3 in $CD4^{+}$T cells and $CD8^{+}$T cells isolated from the peripheral blood of RTP patients, convalescent patients, and healthy controls.

(**C**) Representative density plots and percentage statistics calculated for TIM-3 in gated $CD56^{+}$NK cells.

From A to C, n = 30 for RTP patients, n = 10 for convalescent patients, and n = 10 for healthy controls. All data were analyzed by two-way ANOVA. $*P < 0.05$. Data are presented as mean ± SD.

Sfig.3

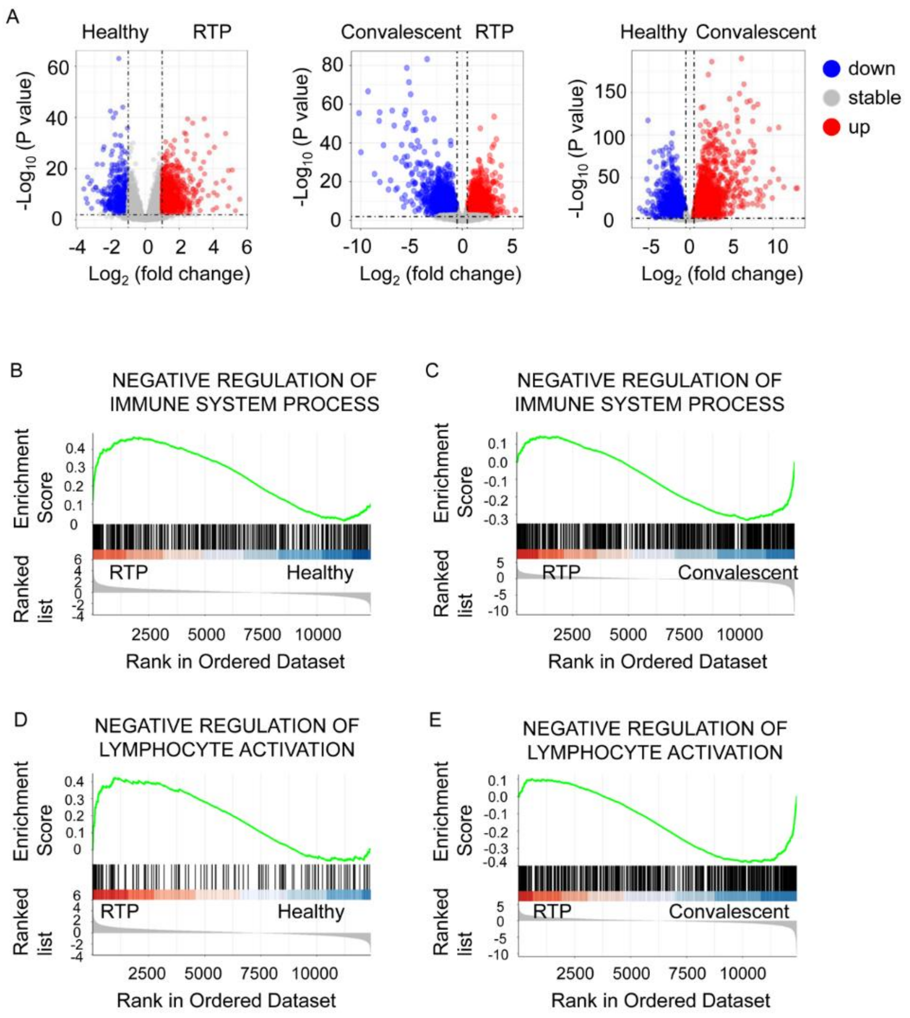

Supplementary Figure 3. Negative regulatory pathways of immune-system processes and lymphocyte activation are enriched in RTP patients.

(**A**) Volcano plot of genes with differential expression in RTP patients compared with that in convalescent patients and healthy controls, and in healthy controls compared with that in convalescent patients. The blue spots represent downregulated genes; the red spots represent upregulated genes.

(**B** and **C**) Gene set enrichment analysis (GSEA) revealed an increase in negative regulation of immune-system processes in RTP patients compared with that in healthy controls (**B**) and convalescent patients (**C**).

(**D** and **E**) GSEA revealed an increase in negative regulation of immune-system processes in RTP patients compared with that in healthy controls (**D**) and convalescent patients (**E**).

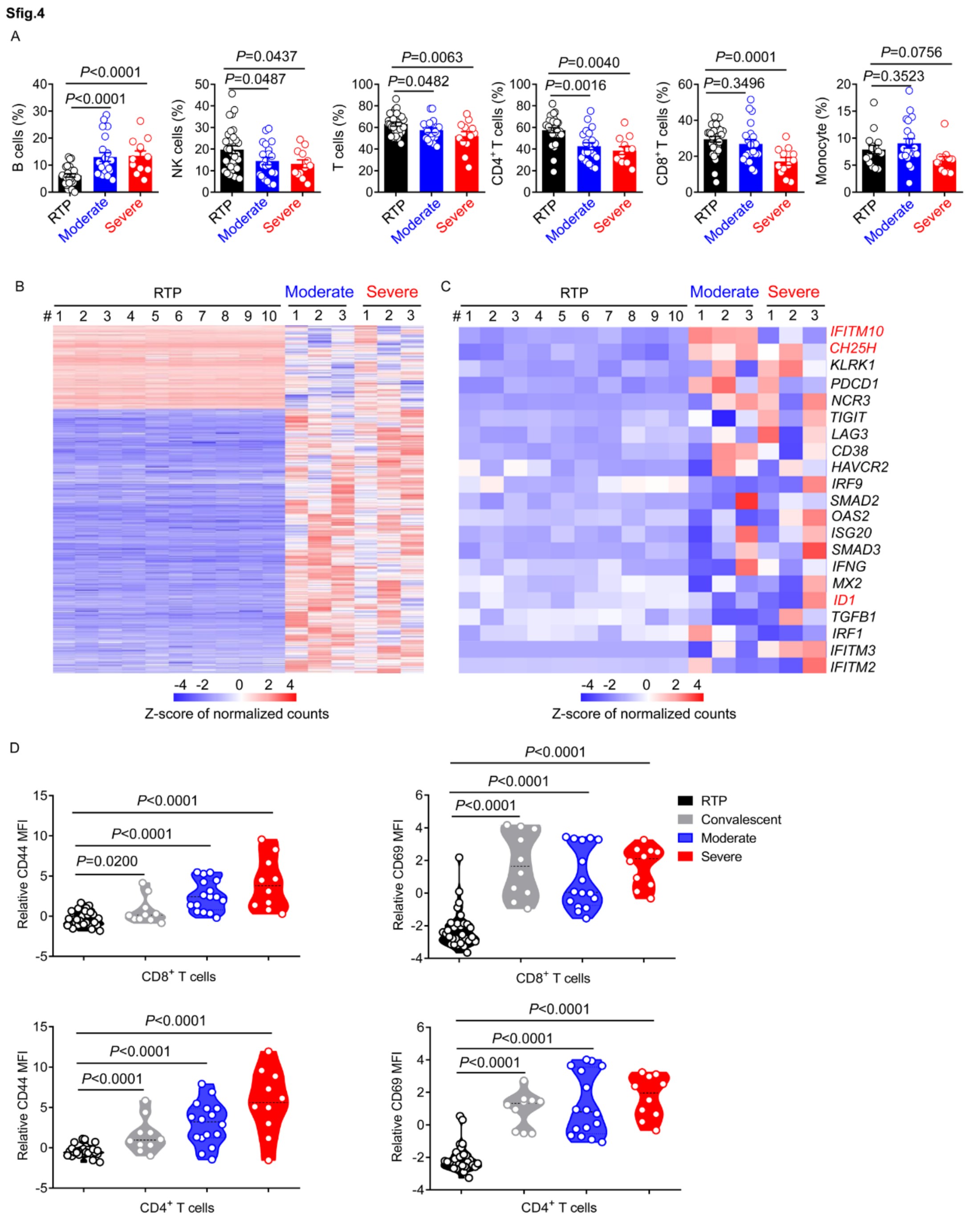

Supplementary Figure 4. Decreased B cells proportion and reduced T cells activation in RTP patients compared with actively infected patients.

(**A**) The frequencies of B cells, NK cells, T cells, CD4^{+}T cells, CD8^{+}T cells and monocytes in RTP patients (black, *n* = 30), patients with moderate COVID-19 (blue, *n* = 21), and patients with severe COVID-19 (red, *n* = 12).

(**B**) Heat map showing differentially-expressed genes (fold change >2, adjusted *P* < 0.05) in RTP patients compared with those in patients with moderate COVID-19, and patients with severe COVID-19. Each column depicts one sample.

(**C**) Heatmap showing the expression of important immune function-related genes in RTP patients compared with those in patients with moderate COVID-19, and patients with severe COVID-19. Each column depicts one sample.

(**D**) Statistical analyses of the relative MFI of CD69 and CD44 in gated NK cells, or $CD45^{+}CD3^{+}CD8^{+}$T cells or $CD45^{+}CD3^{+}CD4^{+}$T cells isolated from the peripheral blood of RTP patients, convalescent patients, patients with moderate COVID-19, and patients with severe COVID-19. The relative MFI was normalized to the MFI of healthy controls. n = 30 for RTP patients, n = 10 for convalescent patients, n = 16 for moderate-COVID-19 patients, and n = 10 for severe-COVID-19 patients. Data were analyzed by two-way ANOVA. $^{*}P < 0.05$; $^{**}P < 0.01$; $^{***}P < 0.001$; $^{****}P < 0.0001$. Data are presented as mean ± SD.

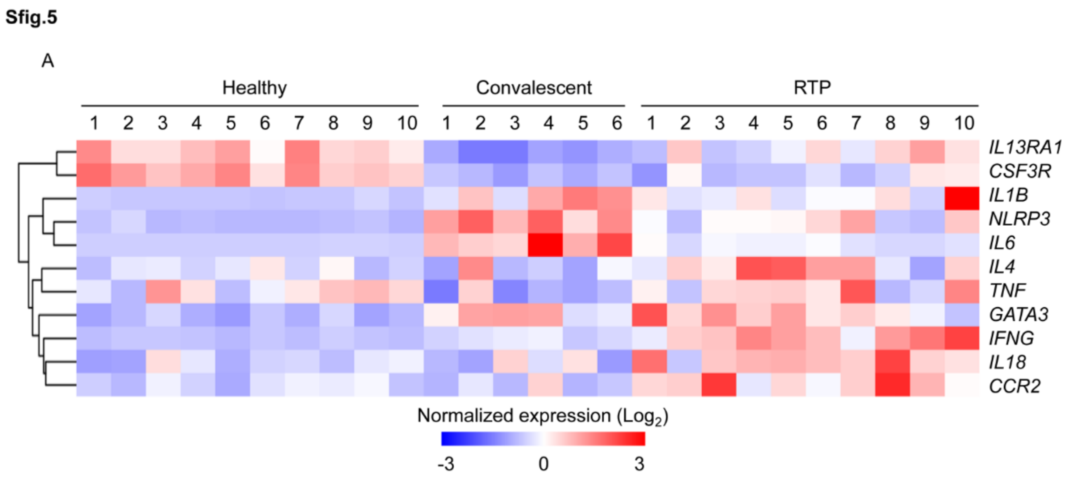

Supplementary Figure 5. Inflammation-related genes show highly expression in the PBMCs from RTP patients.

Heatmap of inflammation-related genes with differential expression in RTP patients relative to that in convalescent patients and healthy controls.

Supplementary Tables

Supplementary Table 1. Baseline characteristics of patients in the study.

Characteristic	Healthy (*n*=20)	Convalescent (*n*=20)	RTP (*n*=30)
Gender (M/F)	12/8	10/10	16/14
Age, years, median (IQR)	67.1 (22–74)	66.7 (32–87)	62 (42–78)
Temperature, ℃, median (IQR)	<37.2	36.5 (36.1–37)	36.6 (36.1–37.1)
White blood cell count, $\times 10^9$/L, median (IQR)	6.73 (3.63–10.06)	8.68 (4.2–24.47)	5.88 (3.37–11.01)
Neutrophil count, $\times 10^9$/L, median (IQR)	4.56 (1.69–8.03)	6.03 (1.96–20.34)	3.60 (1.36–7.29)
Monocyte count, $\times 10^9$/L, median (IQR)	0.48 (0.24–0.75)	0.69 (0.23–1.24)	0.46 (0.16–0.74)
Eosinophil count, $\times 10^9$/L, median (IQR)	1.55 (0.89–2.36)	1.54 (0.56–2.65)	1.57 (1.02–2.31)
Basophil count, $\times 10^9$/L, median (IQR)	0.12 (0.01–0.6)	0.41 (0.01–1.43)	0.23 (0.04–1.03)
Haemoglobin, g/L, median (IQR)	0.02 (0.01–0.04)	0.03 (0.01–0.13)	0.02 (0.01–0.05)
HCT, %, median (IQR)	131.85 (92–161)	98.1 (64–141)	127.78 (90–156)
LDH, U/L,, median (IQR)	39.37 (29.3–47)	29.65 (20.4–41.3)	37.12 (28–46.1)
Neutrophil, %, median (IQR)	229.22 (111–892)	281.55 (144–555)	180.25 (131–312)
Monocyte, %, median (IQR)	65.26 (40.7–81.9)	65.25 (46.4–83.1)	59.38 (37.5–77.4)
Lymphocyte, %, median (IQR)	7.28 (4.5–9.5)	8.28 (4.4–13.8)	7.90 (4.3–16.6)
Eosinophil, %, median (IQR)	25.2 (11.5–47.6)	21.78 (6.3–43.8)	28.25 (16–46.9)
Basophil, %, median (IQR)	2.04 (0.1–9)	4.25 (0.1–12.6)	4.04 (0.3–25.3)

Supplementary Table 2. Antibodies used in flow cytometric analysis.

Antibody	Brand	Cat. No.
Alexa-488 Anti-human CD56	BD Pharmingen	557699
FITC Anti-human CD16	BD Pharmingen	555406
PE Anti-human PD1	BD Pharmingen	560795
APE nti-human CD44	BD Pharmingen	555479
PerCP-CY5.5 Anti-human CD3	BD Pharmingen	300328
PE-CY7 Anti-human CD19	BD Pharmingen	557835
PE-CY7 Anti-human CD56	BD Pharmingen	557747
PE-CY7 Anti-human CD69	BD Pharmingen	557745
PE-CY7 Anti-human NKG2D	Biolegend	320812
APC Anti-human TIM3	eBioscience	17-3109
APC Anti-human CD8	BD Pharmingen	555369
Alexa-647 Anti-human NKp30	BD Pharmingen	558408
Alexa-647 Anti-human CD16	BD Pharmingen	557710
Alexa-647 Anti-human Granzyme B	BD Pharmingen	560212
APC-CY7 Anti-human CD4	BD Pharmingen	557871
V500 Anti-human CD45	BD Pharmingen	560777

No., number; Cat., catalog.

Supplementary Table 3. Main symptoms of RTP patients in the study.

Symptoms, n (%)	RTP (*n*=30)
Fever	0 (0)
Cough	8 (26.6)
Chest tightness	3 (10)
Fatigue	4 (13.3)
